# Research designs and instruments to detect physiotherapy overuse of low-value care services in low back pain management: a scoping review

**DOI:** 10.1186/s12913-023-09166-4

**Published:** 2023-02-23

**Authors:** Lukas Kühn, Lara Lindert, Paulina Kuper, Robert Prill, Kyung-Eun (Anna) Choi

**Affiliations:** 1 Center for Health Services Research, Seebad 82/83, 15562 Rüdersdorf Bei Berlin, Brandenburg Medical School, Neuruppin, Germany; 2 Faculty of Health Sciences Brandenburg, Brandenburg Medical School, Fehrbelliner Straße 38, 16816 Neuruppin, Germany; 3 Center of Orthopaedics and Traumatology, Universtiy Hospital Brandenburg/Havel, Brandenburg Medical School, Neuruppin, Germany; 4 Health Services Research, Faculty of Medicine/Dentistry, Danube Private University, Steiner Landstraße 124, 3500 Krems-Stein, Austria

**Keywords:** Appropriateness of care, Health services overuse, Medical overuse, Physical therapy

## Abstract

**Background:**

The provision of low-value physiotherapy services in low back pain management is a known but complex phenomenon. Thus, this scoping review aims to systematically map existing research designs and instruments of the field in order to discuss the current state of research methodologies and contextualize results to domains and perspectives of a referred low-value care typology. Ultimately, results will be illustrated and transferred to conditions of the German health care setting as care delivery conditions of physiotherapy in Germany face unique particularities.

**Methods:**

The development of this review is guided by the analysis framework of Arksey and O'Malley. A two-stage, audited search strategy was performed in Medline (PubMed), Web of Science, and google scholar. All types of observational studies were included. Identified articles needed to address a pre-determined population, concept, and context framework and had to be published in English or German language. The publication date of included articles was not subject to any limitation. The applied framework to assess the phenomenon of low-value physiotherapy services incorporated three domains (care effectiveness; care efficiency; patient alignment of care) and perspectives (provider; patient; society) of care.

**Results:**

Thirty-three articles met the inclusion criteria. Seventy-nine percent of articles focused on the appropriateness of physiotherapeutic treatments, followed by education and information (30%), the diagnostic process (15%), and goal-setting practice (12%). Study designs were predominantly cross-sectional (58%). Data sources were mainly survey instruments (67%) of which 50% were self-developed. Most studies addressed the effectiveness domain of care (73%) and the provider perspective (88%). The perspective of patient alignment was assessed by 6% of included articles. None of included articles assessed the society perspective. Four methodical approaches of included articles were rated to be transferrable to Germany.

**Conclusion:**

Identified research on low-value physiotherapy care in low back pain management was widely unidimensional. Most articles focused on the effectiveness domain of care and investigated the provider perspective. Most measures were indirectly and did not monitor low-value care trends over a set period of time. Research on low-value physiotherapy care in secondary care conditions, such as Germany, was scarce.

**Registration:**

This review has been registered on open science framework (https://osf.io/vzq7khttps://doi.org/10.17605/OSF.IO/PMF2G).

**Supplementary Information:**

The online version contains supplementary material available at 10.1186/s12913-023-09166-4.

## Background

Across the globe, societies face economic pressures to organize health systems as multidimensional strains impact resource availability. In fact, pandemic conditions, climate change, refugee movements, wars and economic recession have evolved to sustaining challenges of the modern world [1–3]. Considering this given context, it seems compulsory to provide health care which is not only effective, but efficient and in alignment with patient preferences. Addressing this topic, research on the appropriateness of provided health services has gained momentum. In the year of 2018 alone, 839 studies investigated the appropriateness of medical care and provided a data basis indicating a reflection of current practice in almost any area of healthcare [4]. Beyond, specific programs such as “Choosing Wisely” have been developed to actively conquer these trends [5].

Before right care can be emphasized, mechanisms of wrong care need to be uncovered. In that regard, concepts such as medical overuse or underuse as well as low-value care have been defined to approach this phenomenon [6]. For this review, the concept of low-value care was applied which broadly describes that the added costs of an intervention do not provide proportional added benefits [7]. In this respect the typology of Verkerk et al. [8] was applied to approach the phenomenon of low-value care as to the authors knowledge, this typology most comprehensively addresses associated areas of low-value care interventions. The typology describes low-value services by dimensions and perspectives of care: From a clinician perspective, ineffective care lacks of evidence, has the potential to cause harm and its benefits do not outweigh its risks. From a societal perspective, inefficient services potentially produce preventable costs as treatment frequencies and volumes exceed empirical recommendations. From a patient perspective, misaligned health services may be effective and efficient but are inconsistent with patients’ preferences and values [8, 9]. Despite the complexity to holistically evaluate low-value care phenomena, individual perspectives (clinician vs. patient) and domains (effective care vs. patient aligned care) can also be positioned in contradiction to another.

Within the effectiveness domain, examples of low-value PT services in acute low back pain (LBP) management include the delivery of exercise therapy, electrotherapy, massage and the recommendation of bed rest in place of endeavours of reassurance and the advice to remain active [10, 11]. In chronic LBP management, examples of low-value PT services incorporate the delivery of electrotherapy, kinesio-taping and back schools in place of exercise therapy combined with educational components following cognitive behaviour therapy approaches [11, 12].

Estimates of the Canadian Institute for Health Information indicate that 30% of provided health services generate no added patient value and potentially cause harm [13]. For instance, 22% to 38% of diagnostic imagery procedures are considered to be incidentalomas [4]. In this regard, diagnostic testing for unspecific LBP represents a major cause of this deficiency [14]. Specifically, a meta-analysis conducted in 2018 identified that one in four LBP patients receive inappropriate imaging services [15]. Considering treatment procedures, the Care Track study revealed that 28% of LBP treatments delivered in Australia were discordant to clinical guideline recommendations [16].

Despite inappropriate services delivered by medical specialists such as general practitioners and orthopaedists, physiotherapists have a unique responsibility in LBP management. Their role incorporates a primary agency responsible for conservative treatment approaches and ongoing support within the rehabilitation process. However, 35% of physiotherapeutic (PT) services delivered for musculoskeletal health conditions are estimated to be discordant with guideline recommendations [17]. Looking at LBP, 28% of PT services are estimated to be discordant with recommendations [17].

The detection of such trends brings along methodological insecurities in any area of interest. Considering PT services, many health systems fail to systematically collect PT relevant patient level data, prohibiting the appropriate contextualization of diagnostic or treatment selection procedures [9]. Direct measurement approaches, such as systematic evaluations of medical registries are potentially incomplete as many health care settings fall behind digitalization standards. Contrary, indirect measures, such as potential detections of regional variations [9] fail to address contextual conditions allowing a reasonable interpretation of available data.

In addition, the field of physiotherapy is determined by various professional legislations which can strongly differ between countries [18]. These particularities can be illustrated by the German example: To have access to PT care, physicians incorporate a gatekeeper role to PT services. They assess and diagnose patients, before prescribing the number, volumes and the intended PT services to be provided [19].

Regularly, one prescription contains of six treatment sessions [20]. Prescribed services are organized in interventional groups containing of functional exercise therapy, manual therapy, massage therapy, neurological therapy, or resistance training to name most commonly delivered groups [20]. Within these groups, physiotherapists are given room to shape treatment approaches. Nevertheless, they rely on physicians’ assessments to decide whether therapy ends or will be prolonged [19].

To be specific, German physiotherapists are prohibited to diagnose introduced patients (including diagnostic imagery), to offer invasive therapy approaches, to use manual therapy techniques extending gentle joint mobilisation, to decide on the appropriateness of PT intervention groups, and to decide on the duration and scope of therapy [20]. These legislative particularities challenge the applicability of research methodologies fitting other healthcare settings. For instance, many countries of the Commonwealth developed legislative conditions, in which PT care is an established health service of primary care [18]. This condition allows different and more importantly, extended approaches to investigate the phenomenon of PT overuse. Despite a likelihood that political influencers have different foci of interest, the illustrated difficulties of developing research methodologies aiming at evaluating the appropriateness of PT services may be one reason why inadequate data availability of current practice patterns of PT care in Germany is still present.

Thus, this scoping review aims to contribute to the field of health services research in physiotherapy care for LBP management by systematically mapping existing research designs and instruments of the field. Thereby, methodological disadvantages of each method can be discussed and contextualized to the domains and perspectives of the referred overuse typology. Ultimately, future research can be guided in the selection of appropriate methodologies addressing the particularities of each individual health system. Explicit research questions are stated as follows:

1. How are low value PT services in LBP management being measured?
2. To what extent are domains and perspectives of the applied low-value care typology equally approached and represented?
3. Which research approaches fit the legal conditions of the German healthcare system?

## Methods

This study was conducted by a multidisciplinary team with proven expertise in clinical PT, health services research and rehabilitation sciences. The design of the scoping review is following the Arksey and O’Malley framework [21] and comprises five consecutive steps: (I) Identification of the research question(s), (II) identification of relevant studies, (III) study selection, (IV) data charting, and (V) compiling and reporting of results. With regards to research question(s) development, the population-concept-context (PCC) framework which is recommended by the Joanna Briggs Institute’s (JBI) Manual for Evidence synthesis was applied [22]. Study execution is reported in concordance to the PRISMA checklist for Scoping Reviews (PRISMA ScR) [23] which is provided as supplement 1. To ensure research transparency, the protocol of this review has been registered on Open Science Framework and was additionally published elsewhere [24, 25].

### Eligibility criteria

The eligibility criteria of this review are introduced by the structure of the PCC-framework and were extended by the additional domain of “types of evidence”. The population was determined by the diagnosis of unspecific LBP. According to classifications of time or severity of pain, all stadiums of disease progression (acute, sub-acute, chronic and recurrent) and all grades of chronic pain severity established by von Korff et al. [26] were included. Regarding the concept of low-value care, relevant studies needed to address one or more aspects of low-value PT service provision in LBP management. Additionally, studies of all sectors of PT care (inpatient, outpatient, and rehabilitative care settings) from all regions in the world were included. Eligible study designs were limited to observational designs as the authors aimed at identifying studies being conducted under real world conditions avoiding experimental study design elements due to reasons of potential distortion. If applicable, available preprint studies and grey literature such as health insurance reports or other reports of governmental entities were enclosed. Opinion paper, editorials, commentaries and case reports were excluded as these article types do not provide requested information on originally conducted research designs and instruments. Moreover, experimental study designs were excluded, as the aim of this review was not to provide information about interventional studies addressing solutions for PT overuse in LBP management. A comprehensive overview of eligibility criteria is provided in Table 1.

**Table 1:** Population Concept Context-framework

Criteria	Characteristics
Population	- All stadiums of unspecific LBP conditions
Concept	- All studies aiming to detect of medical overuse of physiotherapy care in low back pain management regarding effectiveness, treatment efficacy and alignment of care
Context	- Physiotherapy care across all sectors of health services (inpatient, outpatient and rehabilitation healthcare settings)
Types of evidence	- All types of observational studies - Studies across all countries - Articles published in English or German language - Published and unpublished studies

### Search strategy

A systematic search for eligible studies was conducted up until November 24^th^, 2021. Included databases for the selection of eligible studies were Medline (PubMed), Web of Science and Google Scholar. The search strategy contained keywords and subject headings from referring domains of the PCC framework. Within domains, keywords and index terms were combined with the Boolean operator “OR”. If applicable, keywords and index terms were truncated. To connect domains, the operator “AND” was applied. The research team followed a two-step study selection approach. In step one, an initial, limited search of set databases with predefined keywords was conducted. Search results were used to screen retrieved articles for additional keywords and index terms. In step two, a second search including all identified keywords and index terms was performed. A comprehensive illustration of the search strategy is provided in Additional file 2. To ensure high quality in the study selection process, the search strategy was additionally guided by the Peer Review of Electronic Search Strategies (PRESS) checklist [27] which is provided in supplement 3.

The screening process of retrieved articles was performed in two consecutive steps: In step one, two reviewers (LK, LL) conducted a consensus guided title and abstract screening. After independently screening retrieved articles, disagreements between LK und LL were discussed and agreed upon. Due to mutual agreements within the discussion of conflicting studies, consulting a third reviewer was not required. In step two, a full text screening was conducted following the principles of step one in order to identify the final number of included articles. The study selection process was managed by the online application RAYYAN Version 2021 (Cambridge, USA).

### Data extraction and charting

A data charting table was developed by LK and PK and pilot-tested by randomly selecting three of the included articles. Adaptations to the charting table were discussed by consensus between LK and PK. The final charting table included five categories. (I) Publication details: author(s), year, country, type of PT access (II) study details: aim(s), design, data source(s), mode of delivery, population, recruitment, sample size, diagnosis, PT service(s) (III) theory and framework: low-value care association, framework association, domain of low-value-care, perspective of low-value-care (IV) psychometric properties: instrument description, quality assessment, analysis, direct versus indirect measurement of low-value care (V) Challenges and recommendations: limitations, recommendations.

On the basis of this charting table, results were further synthesized and narratively illustrated in additional tables and figures of which each aimed at addressing one of the stated research questions. As the nature of a scoping review is to map but not appraise existing evidence on a scientific field of interest, the research team resigned to provide a critical appraisal of included studies. However, regarding research question three, a three-tier grading table was developed to assess the appropriateness of applied research approaches to PT conditions in Germany. On this matter, influencing variables affecting the transferability of approaches were pre-determined via consensus (LK, PK). They encompassed legislative access conditions to PT care, investigated treatment procedures as well as utilized data sources. A precise operationalization of influencing variables is provided in supplement 4.

## Results

### Literature search

**Fig. 1:**
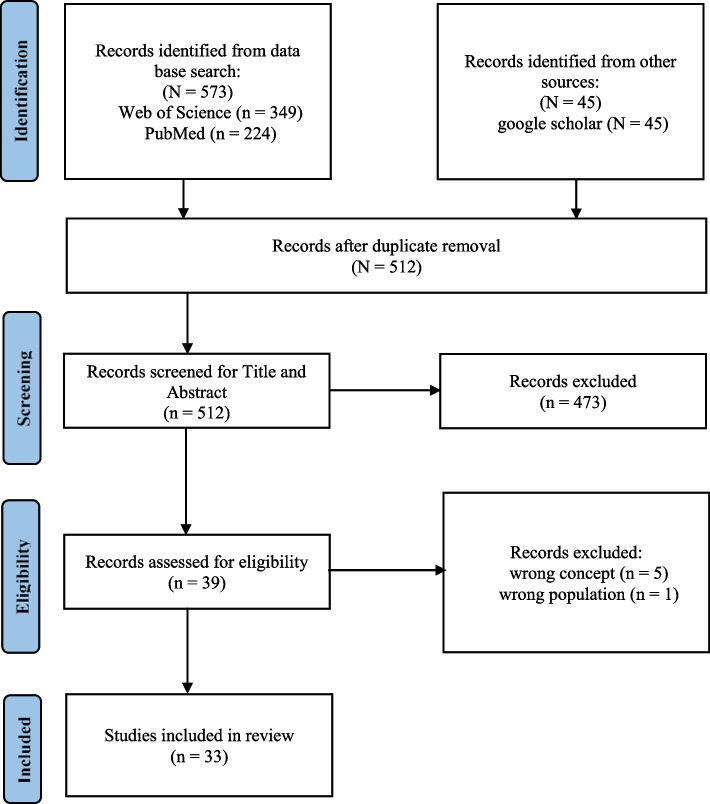
Study selection process according to PRISMA

The initial search was conducted on the electronic databases of Medline (PubMed) and Web of Science and retrieved 348 records. After duplicate removal, 310 records were title and abstract screened to identify additional keywords and index terms for the final search strategy. A list of additionally identified keywords is provided in supplement 2. In the final search, integrated databases were extended by google scholar. In total, 618 articles were identified. After duplicate removal, 512 records were screened for title and abstract of which 39 were assessed for eligibility. According to stated inclusion and exclusion criteria, a final set of 33 articles met the conditions of the literature search. The study selection process is illustrated in Fig. 1.

### Characteristics of included studies

**Fig. 2:**
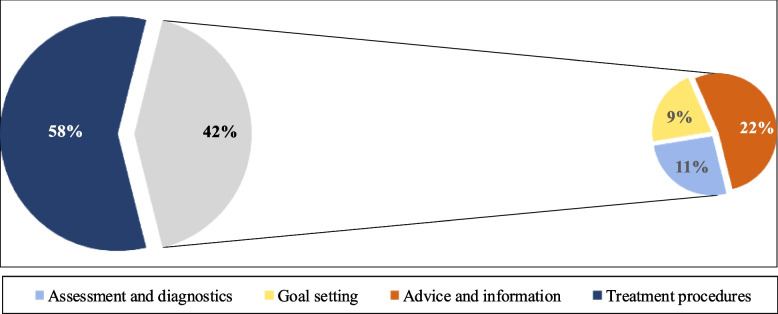
Targeted PT services of included studies Legend: The gray shaded part of the chart contains PT services of “goal setting”, “advice and information” and “treatment procedures”

Included studies were published between 2001 and 2021 and represented 15 countries [28–60]. 30 studies were conducted in western countries [28–32, 35–40, 42–60] of which Canadian studies were most frequently represented (*N* = 5) [39, 50, 56, 57, 60]. Between 2016 and 2021 the highest publication density was identified (*N* = 16) [28–43]. Five studies were conducted in countries with secondary PT care conditions [31, 34, 42, 43, 55]. Figure 2 illustrates targeted PT services of included studies.

Nine associated concepts targeting one or more domains of low-value care were identified. Represented concepts contained ‘current practice patterns’ (*N* = 11) [31, 33, 35, 37–39, 42, 43, 46, 50, 58]; ‘guideline adherence’ (*N* = 8) [32, 34, 40, 41, 45, 48, 54, 55]; ‘clinical management’ (*N* = 6) [49, 51–53, 56, 59]; ‘medical overuse’ (*N* = 2) [36, 47]; ‘utilization’ (*N* = 2) [29, 30]; ‘appropriateness of care’ (*N* = 1) [28], ‘knowledge’ (*N* = 1) [44]; ‘clinical behaviours’ (*N* = 1) [52]; and ‘quality of care’ (*N* = 1) [57].

In 25 studies, the sample population contained physiotherapists [31–36, 38–43, 45, 48, 49, 54, 56, 59, 60]. Of these, five studies included additional occupational groups (osteopaths; chiropractors; general practitioners) [28, 37, 44, 46, 52]. One study included physiotherapists meeting the criteria to be specialized in manual therapy [55]. The remaining eight studies relied on a sample population of LBP patients [29, 30, 47, 50, 51, 53, 57, 58]. Sample sizes varied between 18 and 1361 (median = 219) included cases.

In 18 studies, LBP was not further specified [28–32, 34, 36, 37, 42–46, 48, 51, 54, 58, 59]. Eight studies specifically addressed acute LBP conditions [38, 40, 41, 50, 52, 55, 57, 60]. Six addressed chronic LBP conditions [33, 35, 39, 47, 49, 53], and one study focussed on work-related LBP [56]. Investigated PT services contained treatment procedures (*N* = 27) [28–34, 37–41, 43–45, 47–56, 59, 60]; assessment and diagnostics (*N* = 10) [28, 29, 31, 34, 36, 41, 43, 44, 59, 60], advice and information (*N* = 9) [32, 42, 45, 48, 49, 51, 52, 55, 56]; as well as goal setting practices (*N* = 4) [35, 54, 56, 58]. Detailed study characteristics are provided in Table 2.

**Table 2:** Characteristics of included studies

Reference	Country	PT access	Targeted Concept	Population	Sample Size (N)	LBP diagnosis	Targeted PT services
Wiles, 2022 [28]	Australia	PC	Appropriateness of care	Interdisciplinary experts	20	LBP	Diagnostics, treatment
Singh, 2021 [29]	United Kingdom	PC	Utilization	Patient records	100	LBP	Diagnostics
Licciardone, 2021 [30]	USA	PC	Utilization	Patients	528	LBP	Treatment
Bahns, 2021 [31]	Germany	SC	Current practice patterns	Physiotherapists	1361	LBP	Diagnostics, treatment
Husted, 2020 [32]	Denmark	PC	Guideline adherence	Physiotherapists	234	LBP	Advice, treatment
Alshehri, 2020 [33]	Saudi Arabia	PC	Current practice patterns	Physiotherapists	294	Chronic LBP	Treatment
Akindele, 2020 [34]	Nigeria	SC	Guideline adherence	Physiotherapists	189	LBP	Diagnostics treatment
Gardner, 2018 [35]	Australia	PC	Current practice patterns	Physiotherapists	239	Chronic LBP	Goal-setting
Ely, 2018 [36]	United Kingdom	PC	Medical overuse	Physiotherapists	607	LBP	Diagnostics
Bier, 2018 [37]	Netherlands	PC	Current practice patterns	Physiotherapists & General Practitioners	45	LBP	Treatment
Tumilty, 2017 [38]	New Zealand	PC	Current practice patterns	Physiotherapists	199	Acute LBP	Treatment
Orozco, 2017 [39]	Canada	PC	Current practice patterns	Physiotherapists	846	Chronic LBP	Treatment
Ladeira, 2017 [40]	USA	PC	Guideline adherence	Physiotherapists	528	Acute LBP	Treatment
de Souza 2017 [41]	Brazil	PC	Guideline adherence	Physiotherapists	189	Acute LBP	Diagnostics, treatment
Roussel 2016 [42]	Belgium	SC	Current practice patterns	Physiotherapists	34	LBP	Advice
Billis 2016 [43]	Greece	SC	Current practice patterns	Physiotherapists	154	LBP	Diagnostics, treatment
Ross 2014 [44]	USA	PC	Knowledge	Physiotherapists & General Practitioners	184	LBP	Diagnostics, treatment
Hendrick 2013 [45]	New Zealand	PC	Guideline adherence	Physiotherapists	167	LBP	Advice, treatment
Pincus 2011 [46]	United Kingdom	PC	Current practice patterns	Physiotherapists, Osteopaths, Chiropractors	337	LBP	Advice
Freburger 2011 [47]	USA	PC	Medical overuse	Patients	588	Chronic LBP	Treatment
Rutten 2009 [48]	Netherlands	PC	Guideline adherence	Physiotherapists	472	LBP	Advice, treatment
Liddle 2009 [49]	Ireland	PC	Clinical management	Physiotherapists	419	Chronic LBP	Advice, treatment
Harman 2009 [50]	Canada	PC	Current practice patterns	Patients	164	Acute LBP	Treatment
Casserley-Feeney 2008 [51]	Ireland	PC	Clinical management	Patients	249	LBP	Advice, treatment, waiting times
Bishop 2008 [52]	United Kingdom	PC	Clinical behaviours	Physiotherapists & General Practitioners	1022	Acute LBP	Diagnostics, advice, treatment
Liddle 2007 [53]	United Kingdom	PC	Clinical management	Patients	18	Chronic LBP	Treatment
Swinkels 2005 [54]	Netherlands	PC	Guideline adherence	Physiotherapists	90	LBP	Goal-setting, treatment
Strand 2005 [55]	Norway	SC	Guideline adherence	Manual Therapists	42	Acute LBP	Advice, treatment
Poitras 2005 [56]	Canada	PC	Clinical Management	Physiotherapists	222	Work-related LBP	Goal-setting, advice, treatment
Azoulay 2005 [57]	Canada	PC	Quality of care	Patients	38	Acute LBP	Not applicable
Schonstein, 2002 [58]	Australia	PC	Current practice patterns	Patients	219	LBP	Goal-setting
Gracey 2002 [59]	United Kingdom	PC	Clinical management	Physiotherapists	157	LBP	Diagnostics, treatment
Li 2001 [60]	Canada	PC	management	Physiotherapists	569	Acute LBP	Diagnostics, treatment

Legend: *PC* Primary care, *LBP* Low back pain

### Research designs and instruments to measure low-value physiotherapy care

Key results of this scoping review are illustrated in Tables 2, 3, 4 and 5 at the end of this section. The study designs of included articles were predominantly cross-sectional (*N* = 19) [30–35, 39–41, 44–49, 52, 58, 60]. Eleven studies used a longitudinal design of which eight were observational [29, 50, 51, 54–57, 59] and three within a cohort-design [36–38]. Additionally, two mixed-methods approaches [42, 43], one Delphi expert panel methodology [28] and one observational focus group approach was represented [53]. In line with study designs, data sources were mainly survey data (*N* = 22) [31–35, 37, 39–49, 52, 56, 57, 59, 60] which were followed by patient records (*N* = 4) [29, 50, 51, 58]. Further data sources comprised clinical practice guidelines [28], registry data [30, 54], clinical observations [36, 55], practice management data [38], audio-taped consultations [42], as well as transcripts of qualitative data [43, 49, 56].

**Table 3:** Methodological approaches of included studies

**Reference**	**Study Design**	**Data source**	**Instrument**	**Validation and quality assessment**	**Analysis**	**LVC measure**
Wiles, 2022 [28]	Delphi expert panel	CPGs	N/A	Consensus procedure	Modified three round e-Delphi process	Direct
Singh, 2021 [29]	Longitudinal observational	Patient records	SFÖQ	Validated score	Descriptive analysis	Direct
Licciardone, 2021 [30]	Cross-sectional	Registry data	N/A	N/A	Mc Nemar’s test	Direct
Bahns, 2021 [31]	Cross-sectional	Survey	Self-developed	Pilot-test; plausibility check	Descriptive analysis with 80% threshold for “good” adherence	Indirect
Husted, 2020 [32]	Cross-sectional	Survey	Clinical vignettes	Validated by clinicians	#Descriptive analysis #Chi Square test	Indirect
Alshehri, 2020 [33]	Cross-sectional	Survey	Self-developed	Multiple survey revision	Descriptive analysis	Indirect
Akindele, 2020 [34]	Cross-sectional	Survey	Self-developed	N/R	Descriptive analysis with 50% threshold for “good” adherence	Indirect
Gardner, 2018 [35]	Cross-sectional	Survey	Self-developed	Pilot-test	Variance analysis	Indirect
Ely, 2018 [38]	Cohort-study	Clinical observation	Pre-defined clinical assessment variables	Evidence and clinical opinion	Mixed effects logistic regression model	Direct
Bier, 2018 [37]	Cohort-study	Survey	STarT Back Screening Tool	N/A	Descriptive analysis	Indirect
Tumilty, 2017 [38]	Cohort-study	Practice management database	Treatment summary form	N/A	Descriptive analysis	Direct
Orozco, 2017 [39]	Cross-sectional	Survey	Clinical vignettes	Validated by clinicians	#Descriptive analysis #Chi Square test	Indirect
Ladeira, 2017 [40]	Cross-sectional	Survey	Clinical vignettes	Validated by clinicians	Descriptive analysis	Indirect
de Souza, 2017 [41]	Cross-sectional	Survey	Clinical vignettes	Validated by clinicans	Descriptive analysis	Indirect
Roussel, 2016 [42]	Mixed-methods	Audio-taped consultation & survey	Leventhal’s Common Sense Model	N/R	Descriptive content analysis	Direct
Billis, 2016 [43]	Mixed-methods	Focus group & survey	Self-developed	N/R	#Descriptive analysis #Chi Square test	Indirect
Ross, 2014 [44]	Cross-sectional	Survey	Pre-developed clinical examinations	N/R	Relative Risk Ratio	Indirect
Hendrick, 2013 [45]	Cross-sectional	Survey	Self-developed	Expert opinion and pilot-testing	Logistic Regression analysis	Indirect
Pincus, 2011 [46]	Cross-sectional	Survey	Self-developed	Items based on previously conducted interview study	#Descriptive analysis #Factor analysis	Indirect
Freburger, 2011 [47]	Cross-sectional	Survey	Self-developed	N/R	Bivariate and multivariate analyses	Indirect
Rutten, 2009 [48]	Cross-sectional	Survey	Clinical Vignettes	Theory-based survey (GUIDE Framework); pre-test; Validated by clinicians	#Descriptive analysis #Pearson’s correlation	Indirect
Liddle, 2009 [49]	Cross-sectional	Survey	Self-developed	Pilot-test	Descriptive analysis	Indirect
Harman, 2009 [50]	Longitudinal observational	Patient records	N/A	Interrater agreement; focus groups to validate audit results	Descriptive analysis	Direct
Casserley-Feeney, 2008 [51]	Longitudinal observational	Patient records	N/A	Face validity ensured by pilot testing of 10 charts	#descriptive analysis #Chi Square and t-test	Direct
Bishop, 2008 [52]	Cross-sectional	Survey	Clinical Vignette	Validated by published expert consensus	#descriptive analysis #ANOVA	Indirect
Liddle, 2007 [53]	Observational	Focus groups	Pre-developed focus group guide	Check for internal consistency of coding results	Descriptive content analysis	Indirect
Swinkels, 2005 [54]	Longitudinal observational	Registry data	N/A	N/A	#Descriptive analysis #Chi Square and t-test	Direct
Strand, 2005 [55]	Longitudinal observational	Structured observations & semi-structured interviews	Thematic content analysis	N/R	#Descriptive content analysis	Direct
Poitras, 2005 [56]	Longitudinal observational	Survey	Self-developed	Validated by key informant discussion	#descriptive analysis #regional distribution analysis #Chi Square and t-test	Indirect
Azoulay, 2005 [57]	Longitudinal observational	Survey	Self-developed	Validated by clinicians	Logistic regression analysis	direct
Schonstein, 2002 [58]	Cross-sectional	Patient records	N/R	N/R	Descriptive content analysis	Direct
Gracey, 2002 [59]	Longitudinal observational	Survey	Self-developed	Pilot-study	#descriptive analysis #Chi Square test	Indirect
Li, 2001 [60]	Cross-sectional	Survey	Clinical vignettes	Validated by clinicians	# descriptive analysis # analysis of variance	indirect

Legend: *SFÖQ* Short-Form Örebro Musculosceletal Questionnaire, *CPGs* Clinical practice guidelines, *LVC* Low-value care *N/A* Not applicable, *N/R* Not reported

**Table 4:** Addressed domains and perspectives of low-value care

Reference	Domain			Perspective		
	**Effectiveness**	**Efficiency**	**Patient Alignment**	**Provider**	**Patient**	**Society**
Wiles, 2022 [28]	✓	✓		✓		
Singh, 2021 [29]		✓		✓		
Licciardone, 2021 [30]	✓				✓	
Bahns 2021 [31]	✓			✓		
Husted, 2020 [32]	✓			✓		
Alshehri 2020 [33]	✓			✓		
Akindele 2020 [34]	✓			✓		
Gardner 2018 [35]		✓		✓		
Ely 2018 [36]	✓			✓		
Bier 2018 [37]		✓		✓		
Tumilty 2017 [38]	✓			✓		
Orozco 2017 [39]		✓		✓		
Ladeira 2017 [40]	✓			✓		
de Souza 2017 [41]	✓			✓		
Roussel 2016 [42]	✓			✓		
Billis 2016 [43]		✓		✓		
Ross 2014 [44]	✓			✓		
Hendrick 2013 [45]	✓			✓		
Pincus 2011 [46]		✓		✓		
Freburger 2011 [47]	✓				✓	
Rutten 2009 [48]	✓			✓		
Liddle 2009 [49]	✓			✓		
Harman 2009 [50]	✓	✓		✓		
Casserley-Feeney 2008 [51]	✓	✓		✓		
Bishop 2008 [52]	✓			✓		
Liddle 2007 [53]			✓		✓	
Swinkels, 2005 [54]	✓			✓		
Strand 2005 [55]	✓			✓		
Poitras, 2005 [56]	✓			✓		
Azoulay, 2005 [57]			✓		✓	
Schonstein, 2002 [58]		✓		✓		
Gracey, 2002 [59]	✓	✓		✓		
Li, 2001 [60]	✓			✓		
**Total** **count**	**24**	**11**	**2**	**29**	**4**	**0**

**Table 5:** Transferability to physiotherapy conditions in Germany

Reference	PT access	PT services	Data source
Wiles 2022 [28]	**!**	✓	✓
Singh 2021 [29]	**!**	✓	✓
Licciardone 2021 [30]	**!**	✓	⊗
Bahns 2021 [31]	✓	✓	✓
Husted 2020 [32]	**!**	✓	✓
Alshehri 2020 [33]	**!**	⊗	✓
Akindele 2020 [34]	✓	✓	✓
Gardner 2018 [35]	**!**	✓	✓
Ely 2018 [36]	**!**	⊗	✓
Bier 2018 [37]	**!**	⊗	✓
Tumilty 2017 [38]	**!**	⊗	✓
Orozco 2017 [39]	**!**	**!**	✓
Ladeira 2017 [40]	**!**	⊗	✓
de Souza 2017 [41]	**!**	⊗	✓
Roussel 2016 [42]	✓	✓	✓
Billis 2016 [43]	✓	**!**	✓
Ross 2014 [44]	**!**	✓	✓
Hendrick 2013 [45]	**!**	**!**	✓
Pincus 2011 [46]	**!**	**!**	✓
Freburger 2011 [47]	**!**	✓	✓
Rutten 2009 [48]	**!**	⊗	✓
Liddle 2009 [49]	**!**	**!**	✓
Harman 2009 [50]	**!**	**!**	✓
Casserley-Feeney 2008 [51]	**!**	**!**	✓
Bishop 2008 [52]	**!**	**!**	✓
Liddle 2007 [53]	**!**	✓	✓
Swinkels 2005 [54]	**!**	**!**	⊗
Strand 2005 [55]	✓	✓	✓
Poitras 2005 [56]	**!**	✓	✓
Azoulay 2005 [57]	**!**	✓	✓
Schonstein 2002 [52]	**!**	✓	✓
Gracey 2002 [59]	**!**	**!**	✓
Li, 2001 [60]	**!**	**!**	✓

Legend ⊗: not applicable; **!**: partially applicable; ✓: fully applicable

Instrument measures of survey studies were primarily self-developed (*N* = 11) [31, 33, 34, 43–47, 56, 57, 59]. Authors of seven survey studies applied validated clinical vignettes to measure aspects of care appropriateness [32, 39–41, 48, 52, 60]. Additionally, Bier et al. [37] assessed the prevalence of stratified PT care approaches in the Netherlands by surveying LBP patients with the validated STarT Back Screening Tool [61] to identify psychosocial risk factors and cross checked these findings with self-reported treatment procedures of referring PTs and general practitioners. Another study used pre-developed clinical examinations and referring treatment options to assess differences in knowledge and beliefs between the groups of PTs and general practitioners [44].

With regards to patient records analyses, one study investigated records for psychological, social and lifestyle screening documentation by auditing records on if and which domains of the Short Form Örebro Musculoskeletal Questionnaire (SFÖQ) [62] were documented [29]. Remaining studies using patient records as primary data source applied pre-developed screening frameworks to identify aspects of the patient profile and PT management procedures (e.g. waiting-times; treatment; number and duration of treatment) [50, 51] or the quality of therapy goal documentation in clinical practice [58].

In another study, the PRECISION Pain Research Registry (USA) [63] was used to assess patient-reported PT treatment before and during the early stage of the COVID-19 pandemic [30]. Despite treatment documentation, patients also routinely provided health-status information via validated instruments addressing chronic back pain (Roland-Morris Disability Questionnaire (RMDQ) [64]; 29-item Patient-Reported Outcomes measurement Information System (PROMIS-29) [65], Pain Catastrophizing Scale [66], and Pain Self-Efficacy Questionnaire [67]). The second study using registry data is based on the Dutch registration network for physiotherapists and contained information about patient characteristics, referral information, characteristics of the health problem as well as aspects of the treatment plan which are inserted into the registry within a defined framework [54]. One research group used practice management data to assess applied treatment techniques in LBP management [38]. In this regard, each PT transferred her treatment notes into a summary form containing treatment modalities as well as standard outcome measures.

In two studies, focus groups were used to assess current diagnostic practice patterns [43] and the clinical PT management of LBP patients [53]. Billis et al. [43] conducted three expert focus group rounds in the context of a sequential mixed-methods design, in order to identify key themes associated with LBP diagnostic practice patterns. In a second step, focus group results informed a survey which was rolled out to Greek and British PTs’ in order to detect differences among diagnostic patterns. In contrast, Liddle et al. [53] conducted three stand-alone focus groups with LBP patients in order to identify experiences, opinions and treatment expectations of Irish LBP patients. In both studies, the analysis of focus group data was explorative aiming at identifying key themes associated with referred constructs of interest.

One approach to assess the quality of diagnostic history taking in PT consultation was conducted by Roussel et al. [42] who audio-taped thirty-four PT history taking sessions with non-specific LBP patients. Subsequently, observations were categorized by applying the Leventhal´s Common Sense Model [68] which was cross checked with a self-reported Illness Perception Questionnaire for LBP patients. Another observational approach using a mix of qualitative methods was used by Strand et al. [55] who conducted an initial field observation of the first consultation between PTs and LBP patients followed by a semi-structured interview of a conveniently selected sample of PTs aiming at exploring their clinical interpretation of findings and provided therapeutic procedures.

Wiles et al. [28] conducted a Delphi study to identify quality indicators constituting appropriate LBP care. The panel was composed by twenty musculoskeletal health experts (PTs: *N* = 6) who systematically selected and assessed LBP-associated quality indicators helping to monitor appropriate LBP management. In the three-round Delphi process, a final set of twenty-seven quality indicators addressing diagnostics (*N* = 8), assessment (*N* = 3), acute care (*N* = 5), and ongoing care (*N* = 11) emerged.

With regards to validation and quality assessment endeavours of applied research instruments, self-developed questionnaires were predominantly pilot-tested and validated by clinicians or referred experts of the field. Out of seven survey studies using clinical vignettes, six were self-developed [32, 39–41, 48, 52, 60]. Two studies described the development process [32, 39]. One study used a previously developed and validated vignette [48].

Methods of analysis were predominantly descriptive in nature. Within studies addressing guideline adherence, a percentage threshold which varied between 50 and 80% was set to describe “good” adherence [31, 34]. If applicable, differences between occupational or patient groups were assessed by common test statistics (Chi-Square; Mc Nemar’s; ANOVA; t-test). If collected, patient and clinician characteristics were assessed for explanatory variables of care appropriateness via multivariate analyses (logistic regression analysis; factor analysis). Data of qualitative studies were predominantly analysed using descriptive content analysis. Of included articles, twelve studies investigated aspects of low-value PT service provision via direct measures [28–30, 36, 38, 42, 50, 51, 54, 55, 57, 58]. A detailed display of study characteristics is provided in Table 2. A detailed display of methodical approaches is provided in Table 3.

### Domains and perspectives of low-value care

In accordance to the introduced framework addressing the low-value care phenomenon [8], 24 studies addressed the effectiveness domain by categorically focussing on treatment selection [28, 30–34, 36, 38, 40–42, 44, 45, 47–52, 54–56, 59, 60]. In contrast, eleven studies addressed the efficiency domain by further investigating therapeutic procedures in terms of access, waiting-time, treatment-duration, contents and frequencies [28, 29, 35, 37, 39, 43, 46, 50, 51, 58, 59]. The domain of patient alignment was assessed by two studies focussing on patients’ experiences, opinions and expectations [53, 57]. Regarding perspectives adopted to assess introduced domains, 29 studies targeted healthcare providers as study objective [28, 29, 31–46, 48–52, 54–56, 58–60]. Four studies targeted LBP patients of which two actually incorporated patient perspectives [53, 57] while the other two predominantly targeted patients as a source to reconstruct PT treatment courses [30, 47]. None of included studies targeted a societal perspective as health economic evaluations are pending. The distribution of addressed domains and perspectives across included studies is illustrated in Table 4.

### Transferability to physiotherapy conditions in Germany

Considering pre-determined criteria affecting the transferability of identified research approaches to Germany, four of included studies met all three criteria [31, 34, 42, 55]. Studies meeting secondary care conditions arose from Germany [31], Nigeria [34], Belgium [42], Greece [43], and Norway [55]. Remaining studies were conducted in primary care settings which in accordance were evaluated to limitedly be applicable to German PT care conditions as soon as diagnostics assessments and treatment selection assessments extended authorizations of German physiotherapists. Studies using claims or registry data as a primary data source [30, 54] were graded to be inapplicable as comparable data availability is pending in Germany. A comprehensive illustration of graded transferability criteria is provided in Table 5.

## Discussion

This scoping review mapped existing approaches of research designs and instruments addressing low-value PT care in LBP management. In that respect, the field is currently dominated by indirect measures predominantly relying on cross-sectional study designs using self-developed questionnaires. Moreover, identified studies have largely been conducted under primary care conditions. Over the time of investigation (2001–2021), there was no recognizable trend pointing to preferably applied research methods of particular time periods. However, recent studies additionally used registry data or practice management databases as information sources of direct low-value care measures. Unfortunately, in many PT and healthcare contexts the availability of direct measures is still a goal needing to be achieved. This seems to be particularly relevant for PT conditions under secondary care. In that regard, advancing digitization in PT treatment documentation may allow new opportunities in the exploration of low-value care mechanisms.

Following Chalmers and colleagues [69] who address low-value care mechanisms by the lenses of patient-centric (patient-indication and patient-population lens) versus service-centric (service lens) care, authors of retrieved articles primarily used the patient-indication lens to investigate this phenomenon. This may be attributed to a lacking availability of routinely collected direct measures of physiotherapy care which represents a prerequisite to investigate low-value PT care through the patient-population or the service lens.

Main concepts being targeted as research objectives included current practice patterns of care as well as guideline adherence. Regarding represented domains and perspectives of the low-value care typology, a majority of investigated studies focussed on the effectiveness domain and the provider perspective of care. This finding can become problematic as soon as study-results do not indicate low-value PT care on effectiveness level but fail to identify this phenomenon at the efficiency or patient alignment level as high-value care can only be accomplished if postulated domains remain in harmony.

In that regard, this scoping review offers guidance on methodological considerations by mapping available research designs and instruments and connecting these approaches to domains and perspectives of low value care. Moreover, by using the example of the German PT setting, this review provides a framework on how to map current research approaches to unique legislative conditions of a healthcare setting.

Taking research methodologies and instruments into account, Zadro et al. [17] conducted a systematic review on PT guideline adherence in the management of musculoskeletal conditions. In line with this scoping review, primary measures to assess guideline adherence of LBP management were survey studies with or without clinical vignettes, audits of clinical notes, treatment records or billing codes as well as clinical observations. In that regard, Morgan et al. [70] developed a research agenda to evolve medical overuse research. One of the claims stated in this agenda comprises the development of a national surveillance system of diagnostic and therapeutic frequencies. Taking into account that the detection of low-value PT services is largely measured indirectly, this goal still needs to be achieved. Another aspect of this agenda includes to achieve an agreement of MeSh terms and keywords for electronic database searches in order to identify relevant literature of the field with higher sensitivity [70]. This claim is in line with the authors’ experiences made by conducting this scoping review as the development of the search strategy continually generated new concepts being relevant to low-value care measures.

With respect to identified domains and perspectives of the applied low-value care typology, the question of whether PT care is aligned to patients’ preferences and values was underrepresented in retrieved articles. One concept that addresses to align health care with patients’ needs, is shared decision-making. It encompasses a collaborative process in which caregivers and patients discuss care options under consideration of patients’ individual preferences, values and circumstances [71]. Indeed, studies on the use of shared-decision making procedures in PT practice evolved in recent years [72–74]. Unfortunately, the rationality to evaluate shared-decision making use was predominantly linked to arguments of self-efficacy improvement, patient empowerment, improved clinical communication and less to PT overuse prevention [72–74].

Another challenge of the research on low-value PT care in LBP management goes along with lacking recommendations on efficiency level. For instance, the German National Care Guideline for non-specific back pain provides information about the effectiveness of non-pharmacological therapies but fails to provide information about volumes, intensity, content and other aspects of therapeutic delivery modes [12]. This is reflected by a narrative review of twelve international practice guidelines for low back pain management which identified an inconsistency of recommendations for delivery modes and contents of exercise therapy approaches in acute LBP management [11]. Moreover, indications for some treatment techniques such as spinal manipulation differed across recommendations and authors concluded that current practice guidelines fail to provide cost-effectiveness information to recommended interventions [11].

Regarding exercise efficiency in chronic LBP management, a narrative review of Cashin et al. [75] provides considerations to reflect: Specifically, they give insights about data availability on exercise selection, exercise dose, promotion of exercise participation, supervision, pain management during exercise and the integration of self-management strategies. As the list of aspects having impact on exercise efficiency highlights the complexity to address low-value PT care in LBP management, it will be of relevance to define a common sense of efficient exercise delivery to name only one PT service in order to evolve the research of low-value care in the context of PT services research.

Focussing on a societal perspective of PT care, studies have been addressing the cost effectiveness of PT services in LBP management [76–79]. Nevertheless, these economic evaluations were exclusively part of randomized controlled effectiveness trials and thus, do not allow conclusions about potential economic burdens of contemporary PT practice patterns in day-to-day care.

Generally, research on inappropriate care mechanisms has gained momentum since initiatives such as the Call for Action series of the Lancet Journal put medical overuse in LBP management on the forefront of research communication [80]. Especially, PT Professional Associations from countries like Australia and Brazil have taken action to face overuse trends in PT care by actively engaging into the Choosing Wisely campaign [81, 82]. In Germany however, these trends are pending as the research on the appropriateness of PT services faces significant desiderata [83].

### Strengths and limitations

This scoping review is the first of its kind as it provides insights about the current state of low value care research in PT service provision for LBP patients. It further contextualizes low-value care measures to a framework typology aiming at capturing the phenomenon comprehensively. Regarding the suitability of the applied framework, the authors were able to assign all identified themes and constructs to its domains and perspectives which underlines its comprehensiveness. By rigorously following methodological standards of Arksey and O’Malley [21] as well as the JBI methodology [22], the review adopts a state of the art development process. Furthermore, a two-step search strategy supported the identification of multiple aspects and keywords of low-value PT care. By providing PRISMA-ScR [23] and PRESS [27] reporting checklists a high level of research transparency was achieved.

However, the authors have some limitations to state. Although the applied search strategy followed accepted standards and was peer reviewed by a librarian, the authors cannot assure a comprehensive keyword selection process addressing all aspects of low-value care as the concept still lacks a distinct definition. Referring to this, applied keywords may be subject to a biased study selection. Moreover, this review does not provide a critical appraisal of selected articles, though it is not obligatory for this review methodology [22]. Concerning the methodological approach to transfer review results to legislative PT conditions in Germany, the authors are aware that a three level applicability rating of affecting variables gives space to ambiguity. Thus, this review primarily provides an overview of elementary legal and structural premises fitting German PT conditions. The next step is to clearly operationalize how and which PT services can be investigated at both, the effectiveness- and the efficiency-level in Germany.

## Conclusion

This scoping review provides valuable insights on contemporary research designs and instruments addressing low-value PT care in LBP management. Beyond, it successfully exposes desiderata in PT health services research which has the potential to guide further activities of the field. Identified research on low-value PT care in low back pain management was widely unidimensional. Most articles focused on the effectiveness domain of care and investigated the provider perspective. Most measures were indirectly and did not monitor low-value care trends over a set period of time. Research on low-value physiotherapy care in secondary care conditions, such as Germany, was scarce.

## Supplementary Information

**Additional file 1.**

**Additional file 2**.

**Additional file 3.**

**Additional file 4.**

## Data Availability

All data analysed for this Scoping Review are available in this article and its provided supplements. Ethics approval and consent to participate. Not abblicable.

## Abbreviations

JBI: Joanna Briggs Institute
LBP: Low back pain
PCC: Population, concept, context framework
PT: Physiotherapy
