# Supplementary material for: Research designs and instruments to detect physiotherapy overuse of low-value care services in low back pain management: a scoping review
Source: BMC Health Serv Res. 2023 Feb 23;23:193. doi: 10.1186/s12913-023-09166-4 (PMC9949696; doi:10.1186/s12913-023-09166-4)
Supplement: Supplementary file 2 — Additional file 2. [file 12913_2023_9166_MOESM2_ESM.docx]

**Additional file 2:** Final Search Strategy

Pubmed Nov 24^th^ 2021

| **Concept** | **Results** |
| --- | --- |
| “Low Back Pain”[Mesh] OR “Low Backache*”[all] OR “Postural Low Back Pain”[all] OR “Recurrent Low Back Pain”[all] OR “Lumbago*”[all] OR LBP[all] OR BP[all] OR “acute low back pain”[all] OR ALBP[all] OR “chronic low back pain”[all] OR CLBP[all] OR “sub-acute low back pain”[all] OR backache*[all] OR “other back pain”[all] OR lumboischialgia[all] | 281,441 |
| “Medical Overuse”[Mesh] “Health Services Misuse”[Mesh] OR “health services overutilization”[all] OR “medical preference”[all] OR misdiagnos*[all] OR overdiagnos*[all] OR overuse*[all] OR “overuse* health service*”[all] OR “low-value care”[all] OR overtreatment*[all] OR “inappropriate care”[all] OR “appropriateness of care”[all] OR misuse*[all] OR “misuse of health service*”[all] OR “high-value care”[all] OR “guideline adherence”[all] OR “clinical management”[all] OR “current practice”[all] | 191,656 |
| “Physical Therapy Modalities”[Mesh] OR “physical therapy technique*”[all] OR “physical therap*”[all] OR “physiotherap*”[all] OR “manual therap*”[all] OR “non-invasive therap*”[all] OR “exercise therap*”[all] OR “medical gymnastic*”[all] OR manipulation*[all] OR “spinal manipulation*”[all] OR “physical medicin*”[all] OR physiotherapist*[all] OR “occupational therapist*”[all] OR physio[all] OR “active treatment”[all] OR “exercise treatment”[all] | 439,136 |
| #1 AND #2 AND #3 | 224 |

Web of Science Nov 24^th^ 2021

| **Concept** | **Results** |
| --- | --- |
| "low back pain" (All Fields) or LBP (All Fields) or "back pain" (All Fields) or BP (All Fields) or "acute low back pain" (All Fields) or alba (All Fields) or "chronic low back pain" (All Fields) or clip (All Fields) or "sub-acute low back pain" (All Fields) or lumbag* (All Fields) or backach* (All Fields) or "unspecif* back pain" (All Fields) or "other back pain" (All Fields) or lumboischialgias (All Fields) or "low backach*" (All Fields) or "postural low back pain" (All Fields) or "recurrent low back pain" (All Fields) | 619,272 |
| overus* (All Fields) or "medical overus*" (All Fields) or "overus* service*" (All Fields) or "overus* health service*" (All Fields) or "low-value care" (All Fields) or overtreatment* (All Fields) or overdiagnos* (All Fields) or "inappropriate care" (All Fields) or "appropriateness of care" (All Fields) or misuse* (All Fields) or "misused care" (All Fields) or "misused service*" (All Fields) or "high-value care" (All Fields) or "guideline adherence" (All Fields) or "health service* overuse" (All Fields) or "health service* overutilization" (All Fields) or "medical preference" (All Fields) or misdiagnos* (All Fields) or "current practice" (All Fields) or "clinical management" (All Fields | 140,467 |
| physiotherap* (All Fields) or PT (All Fields) or "physical therap*" (All Fields) or "manual therap*" (All Fields) or "non-invasive therap*" (All Fields) or "exercise therap*" (All Fields) or "medical gymnastic*" (All Fields) or manipulation (All Fields) or "spinal manipulation" (All Fields) or "physical medicin*" (All Fields) or "occupational therap*" (All Fields) or physiotherapist* (All Fields) or "occupational therapist*" (All Fields) or physio (All Fields) or "active treatment*" (All Fields) or "exercise treatment*" (All Fields) or "physical therapy modalit*" (All Fields) or "physical therapy technique*" (All Fields | 701,923 |
| #1 AND #2 AND #3 | 349 |

Google Scholar Nov 24^th^ 2021

| **Concept** | **Results** |
| --- | --- |
| ~”low back pain” | 984,000 |
| ~“medical overuse” | 1,320 |
| ~physiotherapy | 893,000 |
| #1 AND #2 AND #3 | 45 |
