# Supplementary material for: Research designs and instruments to detect physiotherapy overuse of low-value care services in low back pain management: a scoping review
Source: BMC Health Serv Res. 2023 Feb 23;23:193. doi: 10.1186/s12913-023-09166-4 (PMC9949696; doi:10.1186/s12913-023-09166-4)
Supplement: Supplementary file 3 — Additional file 3. [file 12913_2023_9166_MOESM3_ESM.docx]

# Appendix A

***PRESS Guideline* — Search Submission & Peer Review Assessment**

**SEARCH SUBMISSION: THIS SECTION TO BE FILLED IN BY THE SEARCHER**

| Searcher: Kühn, Lukas | Email: | Lukas.kuehn@mhb-fontane.de |
| --- | --- | --- |
| Date submitted: December 5th | Date requested by: | December 8^th^ |

**Systematic Review Title:**

Research designs and instruments to detect physiotherapy overuse of low-value care services in low back pain management: A scoping review

This search strategy is …

| **X** | My PRIMARY (core) database strategy — First time submitting a strategy for search question and database |
| --- | --- |
|  | My PRIMARY (core) strategy — Follow-up review NOT the first time submitting a strategy for search question and database. If this is a response to peer review, itemize the changes made to the review suggestions |
|  | SECONDARY search strategy— First time submitting a strategy for search question and database |
|  | SECONDARY search strategy — NOT the first time submitting a strategy for search question and database. If  this is a response to peer review, itemize the changes made to the review suggestions |

**Database** *[mandatory]*

Medline

**Interface** *[mandatory]*

PubMed

**Research Question**

(Describe the purpose of the search) *[mandatory]*

This scoping review aims to contribute to the field to PT overuse research in LBP management by systematically mapping existing research designs and instruments of the field. Thereby, methodological disadvantages of each method can be discussed and contextualized to the domains and perspectives of the referred overuse typology. Ultimately, future research can be guided in the selection of appropriate methodologies addressing the particularities of each individual health system. Explicit research questions are stated as follows:

1. How is medical overuse of PT services in LBP management being measured?
2. To what extent are domains and perspectives of the applied typology equally approached and represented?
3. Which research approaches fit the legal conditions of the German healthcare system?

**PCC Format**

(Outline the PCCs for your question — i.e., Population, Concept, Context and Study Design — as applicable)

| **Criteria** | **Characteristics** |
| --- | --- |
| **Population** | - All stadiums of unspecific LBP conditions |
| **Concept** | - All studies aiming to detect of medical overuse of physiotherapy care in low back pain management regarding effectiveness, treatment efficacy and alignment of care |
| **Context** | - Physiotherapy care across all sectors of health services (inpatient, outpatient and rehabilitation healthcare settings) |
| **Types of evidence** | - All types of observational studies - Studies across all countries - Articles published in English or German language - Published and unpublished studies |

**Was a search filter applied?**

No

Please copy and paste your search strategy here, exactly as run, including the number of hits per line. ***[mandatory]***

| **Concept** | **Results** |
| --- | --- |
| “Low Back Pain”[Mesh] OR “Low Backache*”[all] OR “Postural Low Back Pain”[all] OR “Recurrent Low Back Pain”[all] OR “Lumbago*”[all] OR LBP[all] OR BP[all] OR “acute low back pain”[all] OR ALBP[all] OR “chronic low back pain”[all] OR CLBP[all] OR “sub-acute low back pain”[all] OR backache*[all] OR “other back pain”[all] OR lumboischialgia[all] | 281,441 |
| “Medical Overuse”[Mesh] “Health Services Misuse”[Mesh] OR “health services overutilization”[all] OR “medical preference”[all] OR misdiagnos*[all] OR overdiagnos*[all] OR overuse*[all] OR “overuse* health service*”[all] OR “low-value care”[all] OR overtreatment*[all] OR “inappropriate care”[all] OR “appropriateness of care”[all] OR misuse*[all] OR “misuse of health service*”[all] OR “high-value care”[all] OR “guideline adherence”[all] OR “clinical management”[all] OR “current practice”[all] | 191,656 |
| “Physical Therapy Modalities”[Mesh] OR “physical therapy technique*”[all] OR “physical therap*”[all] OR “physiotherap*”[all] OR “manual therap*”[all] OR “non-invasive therap*”[all] OR “exercise therap*”[all] OR “medical gymnastic*”[all] OR manipulation*[all] OR “spinal manipulation*”[all] OR “physical medicin*”[all] OR physiotherapist*[all] OR “occupational therapist*”[all] OR physio[all] OR “active treatment”[all] OR “exercise treatment”[all] | 439,136 |
| #1 AND #2 AND #3 | 224 |

**PEER REVIEW ASSESSMENT: THIS SECTION TO BE FILLED IN BY THE REVIEWER**

|  | Reviewer: Lara Lindert | Email: [lara.lindert@mhb-fontane.de](mailto:lara.lindert@mhb-fontane.de) | Date completed: 18.08.2022 | | |
| --- | --- | --- | --- | --- | --- |
|  |  |  |  | | |
|  | **1. TRANSLATION** |  |  | | |
| A -­‐No revisions | | x |  |  |  |
| B -­‐ Revision(s) suggested | | ☐ |  |  |  |
| C -­‐ Revision(s) required | | ☐ |  |  |  |

If “B” or “C,” please provide an explanation or example:

**2. BOOLEAN AND PROXIMITY OPERATORS**

| A -­‐No revisions | x |
| --- | --- |
| B -­‐ Revision(s) suggested | ☐ |
| C -­‐ Revision(s) required | ☐ |

If “B” or “C,” please provide an explanation or example:

**3. SUBJECT HEADINGS**

| A -­‐No revisions | x |
| --- | --- |
| B -­‐ Revision(s) suggested | ☐ |
| C -­‐ Revision(s) required | ☐ |

If “B” or “C,” please provide an explanation or example:

**4. TEXT WORD SEARCHING**

| A -­‐No revisions | x |
| --- | --- |
| B -­‐ Revision(s)suggested | ☐ |
| C -­‐ Revision(s) required | ☐ |

If “B” or “C,” please provide an explanation or example:

**5. SPELLING, SYNTAX, AND LINE NUMBERS**

| A -­‐No revisions | x |
| --- | --- |
| B -­‐ Revision(s)suggested | ☐ |
| C -­‐ Revision(s) required | ☐ |

If “B” or “C,” please provide an explanation or example:

**6. LIMITS AND FILTERS**

| A -­‐No revisions | x |
| --- | --- |
| B -­‐ Revision(s) suggested | ☐ |
| C -­‐ Revision(s) required | ☐ |

If “B” or “C,” please provide an explanation or example:

OVERALL EVALUATION (Note: If one or more “revision required” is noted above, the response below must be “revisions required”.)

| A -­‐No revisions | x |
| --- | --- |
| B -­‐ Revision(s) suggested | ☐ |
| C -­‐ Revision(s) required | ☐ |

Additional comments:
