# Supplementary material for: Research designs and instruments to detect physiotherapy overuse of low-value care services in low back pain management: a scoping review
Source: BMC Health Serv Res. 2023 Feb 23;23:193. doi: 10.1186/s12913-023-09166-4 (PMC9949696; doi:10.1186/s12913-023-09166-4)
Supplement: Supplementary file 4 — Additional file 4. [file 12913_2023_9166_MOESM4_ESM.docx]

**Supplement 4:** Operationalization of influencing variables reflecting on the transferability of retrieved research articles to legislative physiotherapy care conditions in Germany

| **PT access** | |
| --- | --- |
| Fully applicable | Physiotherapy care is established in secondary care. |
| Partially applicable | Physiotherapy care is established in primary care but investigated physiotherapy services can only partially be applied to German physiotherapy care conditions (i.e.: diagnostic imaging services are not covered by the German occupational law of physiotherapy). |
| **PT services** | |
| Fully applicable | Investigated physiotherapy services are practiced and delivered in Germany and are fully transferable to German physiotherapy care conditions. |
| Partially applicable | Only aspects of investigated physiotherapy services are practiced and delivered in Germany as some of them are prohibited by the German occupational law of physiotherapy and thus, cannot fully be transferred to the German setting. |
| Not applicable | None of investigated physiotherapy services are practiced and delivered in Germany and thus, cannot be transferred to German physiotherapy care conditions as the German occupational law of physiotherapy prohibits the usage of investigated services. |
| **Data source** | |
| Fully applicable | The applied data source is available under German physiotherapy care conditions. |
| Not applicable | The applied data source is not available under German physiotherapy care conditions. |
